# A Versatile Method for Determination of Resin Acids in Occupational Softwood Sawmill Dust Samples by Liquid Chromatography‐Atmospheric Pressure Chemical Ionization‐Mass Spectrometry

**DOI:** 10.1002/jssc.70568

**Published:** 2026-11-04

**Authors:** Raymond Olsen, Ingrid Bakke

**Affiliations:** ^1^ Department of Chemical and Biological Work Environment National Institute of Occupational Health (STAMI) Section for Chemical Work Environment Oslo Norway

**Keywords:** isomer resolution, occupational exposure, resin acids, wood dust

## Abstract

Occupational exposure to softwood dust has been linked to upper and lower respiratory symptoms, eye irritation, contact dermatitis, and an elevated risk of sino‐nasal cancer. Resin acids, naturally occurring extractives that vary by tree species, are suspected contributors to these health effects, yet existing analytical methods have struggled to resolve key isomeric acids. This study aimed to develop a rapid, robust, sensitive, and selective liquid chromatography‐mass spectrometry (LC‐MS) method for the determination of individual resin acids in personal wood dust samples from Norwegian sawmills. Six commercially available reversed‐phase columns and multiple mobile phase systems were evaluated for chromatographic performance. Optimal separation was achieved with a C18‐amide column (150 × 2.1 mm, 3 µm) using an isocratic mobile phase of acetonitrile and 30 mM ammonium acetate at pH 6.30, 0.3 mL min^−1^ with a 15‐min runtime. Optimal detection employed atmospheric pressure chemical ionization (APCI) in negative mode. This method was applied to six personal sawmill dust extracts, enabling determination of five resin acids (abietic acid, dehydroabietic acid, levopimaric acid, and the coeluting isopimaric and pimaric acids) and one oxidation product (7‐oxodehydroabietic acid). The LC‐APCI‐MS method improves separation of resin acids over prior LC approaches, with minimal sample preparation, and is suitable for routine monitoring of occupational exposure to resin acids in wood dust.

## Graphical abstract

A versatile, rapid, robust, sensitive, and selective liquid chromatography‐atmospheric pressure chemical ionization‐mass spectrometry method for determining resin acids in wood dust. The method improves isomer resolution compared to previous methods, has minimal sample preparation and a short (15 min) runtime, enabling routine occupational exposure monitoring.

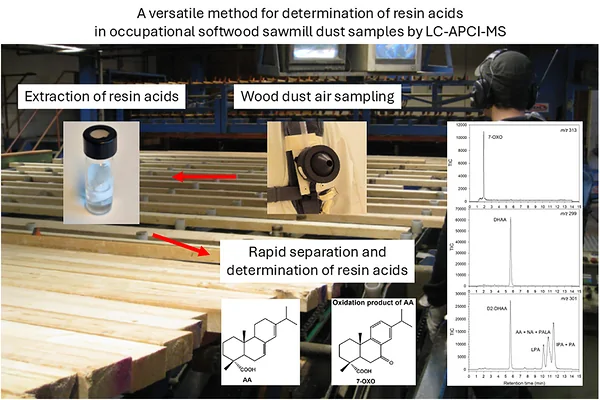

## 1 Introduction

Occupational exposure to dust from softwoods (conifers) has been associated with a wide variety of upper and lower respiratory effects as well as eye irritation and contact dermatitis [1, 2, 3]. Sino‐nasal cancer has also been associated with exposure to softwood dust, although the association with dust from hardwoods (broadleaf trees) is much stronger [4]. Furthermore, wood dust is classified as carcinogenic to humans (group 1) by the International Agency for Research on Cancer (IARC) [4].

Wood is primarily composed of cellulose, polyoses, and lignin, but also contains a variety of lower molecular weight chemicals, referred to as extractives [5]. Extractives have been suspected of causing negative health effects, considering that health effects from wood dust exposure vary between tree species. Resin acids are extractives that are common in many softwoods, and their composition varies considerably by tree species [6]. The softwood resin acids may primarily be divided into two categories: abietane‐type and pimarane‐type. The molecular structures of common resin acids are shown in Figure 1. Resin acids, such as abietic acid (AA), are prone to oxidation when in contact with air [7], and their oxidized forms, primarily 15‐hydroxyperoxyabietic acid (15‐HPA), are considered the main sensitizers of resin acids found in *Pinus* species, conventionally named rosin or colophonium [8, 9, 10]. Hydroperoxides are highly reactive and unstable and thereby difficult to measure accurately. This has resulted in the use of another stable oxidation product of AA, the 7‐oxodehydroabietic acid (7‐OXO), as a marker for oxidation [11].

**FIGURE 1:**
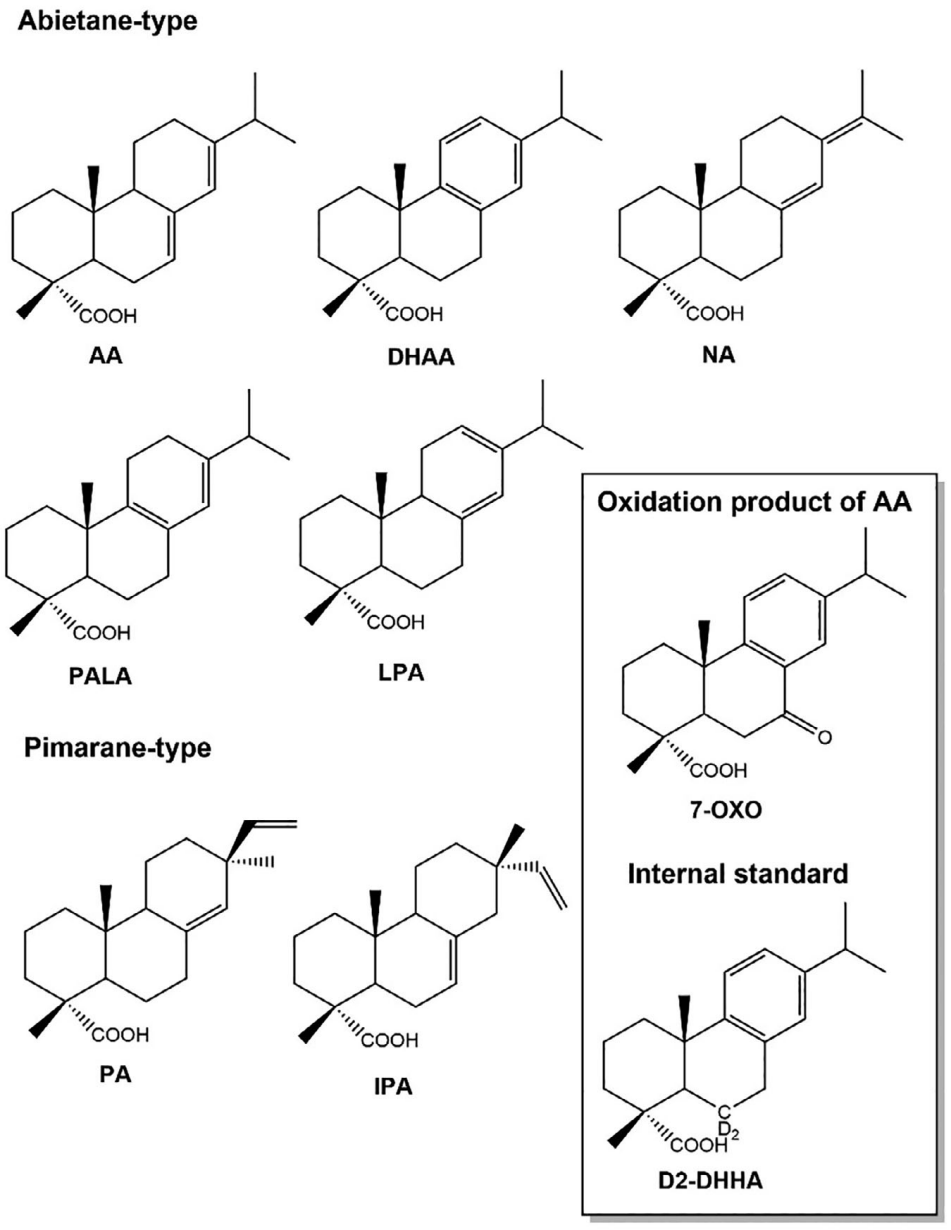
Chemical structure of resin acids, 7‐oxodehydroabietic acid, and internal standard (D2‐DHHA).

Sawmill workers may be occupationally exposed to colophonium, resin acids, and their oxidized derivatives [12, 13]. In Norway, sawmills mainly utilize timber from two different tree species: Scots pine (*Pinus sylvestris*) and Norway spruce (*Picea abies*). These are commonly used in building materials throughout Norway. Thus, sawmill dust exposures in Norwegian sawmills mainly originate from these two wood species. Resin acids are part of the wood matrix, and occupational exposure to resin acids in sawmill facilities has been associated with wood dust exposure. The main resin acid of Scots pine is levopimaric acid (LPA, 30% of total resin acids), followed by approximately equal amounts of AA (15.8%), palustric acid (PALA, 15.1%), and dehydroabietic acid (DHAA, 14.4%) [5]. Norway spruce mainly contains DHAA (22.6%) and LPA (16.2%) [5]. These are all abietane‐type resin acids, which are known to have a stronger fungitoxic effect than pimarane‐type resin acids [14]. Among the pimarane‐ and iso‐pimarane‐type resin acids, Scots pine and Norway spruce both contain pimaric acid (PA) and isopimaric acid (IPA). Scots pine contains PA and IPA approximately at a ratio of 3:1, and Norway spruce at a ratio of 1:2 [5, 14].

Determination of resin acids in different matrices has been performed using a wide range of techniques such as enzyme‐linked immunosorbent assay [15], high‐performance liquid chromatography (HPLC) [8, 16, 17], LC‐atmospheric pressure chemical ionization‐mass spectrometry (LC‐APCI‐MS) [18], LC‐electrospray ionization‐tandem MS (LC‐ESI‐MS/MS) in positive [19, 20] and negative mode [21], and gas chromatography‐MS (GC‐MS) after derivatization to methyl esters [22, 23, 24, 25], perfluorobenzyl esters [26], and trimethylsilyl esters [27, 28]. A GC‐flame ionization detection (GC‐FID) method, which determines the corresponding methyl ethers of the resin acids, is the established method for determination of resin acid concentrations in workroom air [25]. The GC‐FID method has the advantage of high peak resolution, but the derivatization reaction used is time‐consuming and introduces additional sources of analytical error. In addition, the formed methyl esters may easily hydrolyze. Axelsson et al. have presented an alternative method for resin acid analysis without derivatization, using LC‐ESI‐MS in positive mode [20]. The reported LC‐ESI‐MS method provided separation of the two major isomeric resins, PA and AA. However, LPA and IPA were not analytically separated and coeluted prior to AA [20]. As LPA is among the main resin acids of both Scots pine and Norway spruce, there is a need for an LC‐MS method that can provide improved peak resolution of resin acids.

The aim of this study was to develop a versatile, rapid, robust, sensitive, and selective LC‐MS methodology for determination of individual resin acids, including LPA and IPA, and total resin acid in personal softwood dust samples collected during work in Norwegian sawmills.

## 2 Materials and Methods

### 2.1 Chemicals and Materials

LC‐MS grade acetonitrile (ACN) was purchased from Fisher Scientific (Loughborough, UK). HPLC‐grade methanol was obtained from Rathburn Chemicals (Walkerburn, UK). Deionized water was produced using a Milli‐Q Academic water purification system. Analytical grade ammonium acetate (NH_4_Ac) and ammonium formate (NH_4_OOCH) were provided by Merck (Darmstadt, Germany). DHAA (95%) and dehydroabietic acid‐6,6‐d_2_ (d_2_‐DHAA, 95%) were purchased from Ramidus AB (Lund, Sweden). AA (90%–95%), 7‐OXO (>95%), IPA (>99%), LPA (>98%), neoabietic acid (NA, >99%), PA (80%–90%) and PALA (90%–95%) were all obtained from Cansyn Chemical Corp (New Westminster, Canada). Nitrogen gas for sheath and auxiliary gas in the MS was produced using an Inmatec IMT‐PN 1150 nitrogen generator (INMATEC GaseTechnologie GmbH, Herrsching, Germany).

Personal inhalable and thoracic softwood dust samples were collected over 8 h work shifts in Norwegian sawmills using a conical inhalable sampler (CIS; Casella, Bedford, UK) with a 37 mm PVC filter (5 µm pore size; Merck Millipore, Billerica, MA, USA), and a BGI GK2.69 cyclone (BGI Inc., Waltham, MA, USA) mounted with a Millipore 37 mm sampling cassette (Merck Life Sciences, Darmstadt, Germany) and containing a 37 mm PVC filter. The CIS sampler and BGI GK2.69 cyclone were operated with an SKC Universal air sampling pump model 224‐PCXR8, using an airflow of 3.5 L min^−1^, and a Casella TUFF4 PRO IS air sampling pump, using an airflow of 1.6 L min^−1^, respectively.

After gravimetric determination of mass, the 37 mm PVC filter sample was transferred to a 4 mL glass vial with a PTFE/silicone disc screw cap (Thermo Fisher Scientific, San Jose, CA, USA) and stored in a freezer at – 20°C prior to analysis. The sample preparation was performed according to the previously described method (Axelsson et al. [20]), however, with some minor modifications. In brief, the sample was transferred from the freezer to room temperature 1 h before extraction. To the sample, 2 mL of 0.25 µg mL^−1^ d_2_‐DHAA solution in methanol was added and subsequently shaken automatically for 30 min (1500 rpm, Vibramax 110, Heidolph Instruments, Schwabach, Germany). Following extraction, the sample was filtered using a Millex‐GN 0.20 µm 13 mm nylon syringe filter (Merck Millipore). Finally, 8 µL of the sample extract was subjected to LC‐MS analysis.

### 2.2 Instrumentation and Conditions

The LC‐MS system consisted of a Thermo Scientific Dionex Ultimate 3000 RSLC UHPLC system with a binary gradient pump, a thermostatted autosampler, and a thermostatted column compartment connected to a Thermo Scientific TSQ Vantage EMR QqQ‐MS (Thermo Fisher Scientific, Waltham, MA, USA). Six analytical columns with different stationary phases and dimensions were tested in this study; ACE Excel C18 amide (3 µm, 150 mm × 2.1 mm i.d.), ACE C18‐PFP (3 µm, 150 mm × 2.1 mm i.d.), Hypersil Gold (1.9 µm 50 mm × 2.1 mm i.d.), Hypersil Gold‐PFP (1.9 µm 100 × 2.1 mm i.d.), Kromasil 100 C18 (3.5 µm, 150 mm × 2.1 mm i.d.), and Zorbax Bonus‐RP (3.5 µm × 2.1 mm). Mobile phases consisting of ACN and water, with formic acid, ammonium formate, or ammonium acetate to adjust pH, were tested to optimize separation, peak shape, and signal intensity of the selected resin acids. Detection was optimized by testing both positive and negative modes of ESI and APCI. Data generated by the LC‐MS was collected and evaluated using Xcalibur v. 2.2 (Thermo Fisher Scientific).

### 2.3 Optimized Final Method

Chromatographic separation was performed on an analytical column (150 × 2.1 mm I.D. ACE Excel 3 C18‐amide) fitted with a pre‐column filter (ACE UHPLC pre‐column filter) purchased from G&T Septech (Ski, Norway). The isocratic mobile phase consisted of ACN and 30 mM ammonium acetate pH 6.30 (70:30, v/v); the flow rate was 0.3 mL min^−1^, and the total run time was 15 min. The autosampler and column compartment temperatures were set to 20 and 40°C, respectively. Introduction into the MS was done using APCI in negative mode, with a corona needle current of 15 µA, an APCI vaporizer temperature of 343°C, a capillary temperature of 350°C, and an s‐lens rf amplitude of 57 V. Detection and quantification were performed in SIM mode of *m/z* 313.2 (7‐OXO), *m/z* 299.2 (DHAA, LPA, AA, IPA, PA, NA, and PALA) and *m/z* 301.2 (d_2_‐DHAA), corresponding to the deprotonated molecular ions of the resin acids. Dwell time was set to 100 ms. The same analytical approach was briefly described by Straumfors et al. [12, 13]. The analytical approach is described in full detail here to enable routine implementation and repeatability.

## 3 Results and Discussion

### 3.1 Optimization of Separation

The HPLC column with a C12 stationary phase with embedded polar urea groups used by Axelsson et al. [20], which provided separation of PA and AA, had been discontinued by the manufacturer and was thus no longer available from the vendor. Thus, several alternative columns with different stationary phases were tested with respect to separation of resin acids. All columns were initially tested with the same isocratic mobile phase as used by Axelsson et al. [20] (ACN and water (59:41, v/v) with 0.05% formic acid) and a flow rate of 0.3 mL min^−1^. Analytical standards of the resin acids, 7‐OXO, DHAA, IPA, AA, and LPA, were used in the initial testing. However, none of the selected columns provided adequate separation of IPA, AA, and LPA using the selected mobile phase composition. Furthermore, the isocratic mobile phase used by Axelsson et al. did not elute the resin acids from the columns within a 30‐min timeframe. Thus, mobile phase compositions consisting of ACN and water with 5 mM ammonium formate (pH 5.0) or 10 mM ammonium acetate (pH 4.5–6.5) were tested with respect to separation of IPA, AA, and LPA. The separation of the five selected resin acids (7‐OXO, DHAA, IPA, AA, and LPA), with respect to good peak shapes and elution of the resin acids within a 15‐min time frame, was only obtained on the ACE Excel C18 amide column using a mobile phase composition of ACN and 10 mM ammonium acetate (pH 5.5‐6.5) (70/30, v/v). A moderate increase in the separation of IPA, AA, and LPA and an approximately 2‐min decrease in the retention times of IPA, AA, and LPA were observed, with pH increasing from 5.5 to 6.5. To our knowledge, the separation of IPA, AA and LPA has not been accomplished using other LC stationary phases.

### 3.2 Optimization of Detection

The ESI and APCI capability of the resin acids was investigated by direct infusion experiments dissolving the resin acids in ACN and 10 mM ammonium acetate (pH 5.5) (70/30, v/v). Both negative and positive modes were explored, but positive and negative ESI and positive APCI were excluded due to very low ionization efficiency. APCI with negative ionization generated mass spectra, with only the deprotonated molecular ion of the resin acids present. A decrease in signal intensity by a factor of two was observed by increasing the ammonium acetate buffer pH from pH 5.5 to pH 6.5. In this MS method, SIM mode was preferred, as MRM mode yielded no fragment ions of sufficient intensity at the chosen mobile phase composition. The resin acids provided a stable signal with increasing corona needle current from 3 to 15 µA, a vaporizer temperature of 343°C, an ion transfer tube temperature of 350°C, and an s‐lens RF amplitude of 57 V.

To explore the robustness of the chosen mass spectrometric method, 16 consecutive injections using a mobile phase consisting of ACN and 10 mM ammonium acetate (pH 5.5, 6.0, and 6.3) (70/30, v/v) were performed. Considerable losses of the resin acids' signal intensities were observed during these 16 injections using 10 mM ammonium acetate buffer at pH 5.5 and 6.0. In contrast, the mobile phase consisting of ACN and 10 mM ammonium acetate (pH 6.3) (70/30, v/v) provided stability both with regard to signal intensity and retention time, and was thus chosen in the final method. Total ion chromatograms of a calibration solution containing 500 ng mL^−1^ (injection volume of 8 µL) of the resin acids 7‐OXO, DHAA, IPA, AA, and LPA using the optimized method are shown in Figure 2.

**FIGURE 2:**
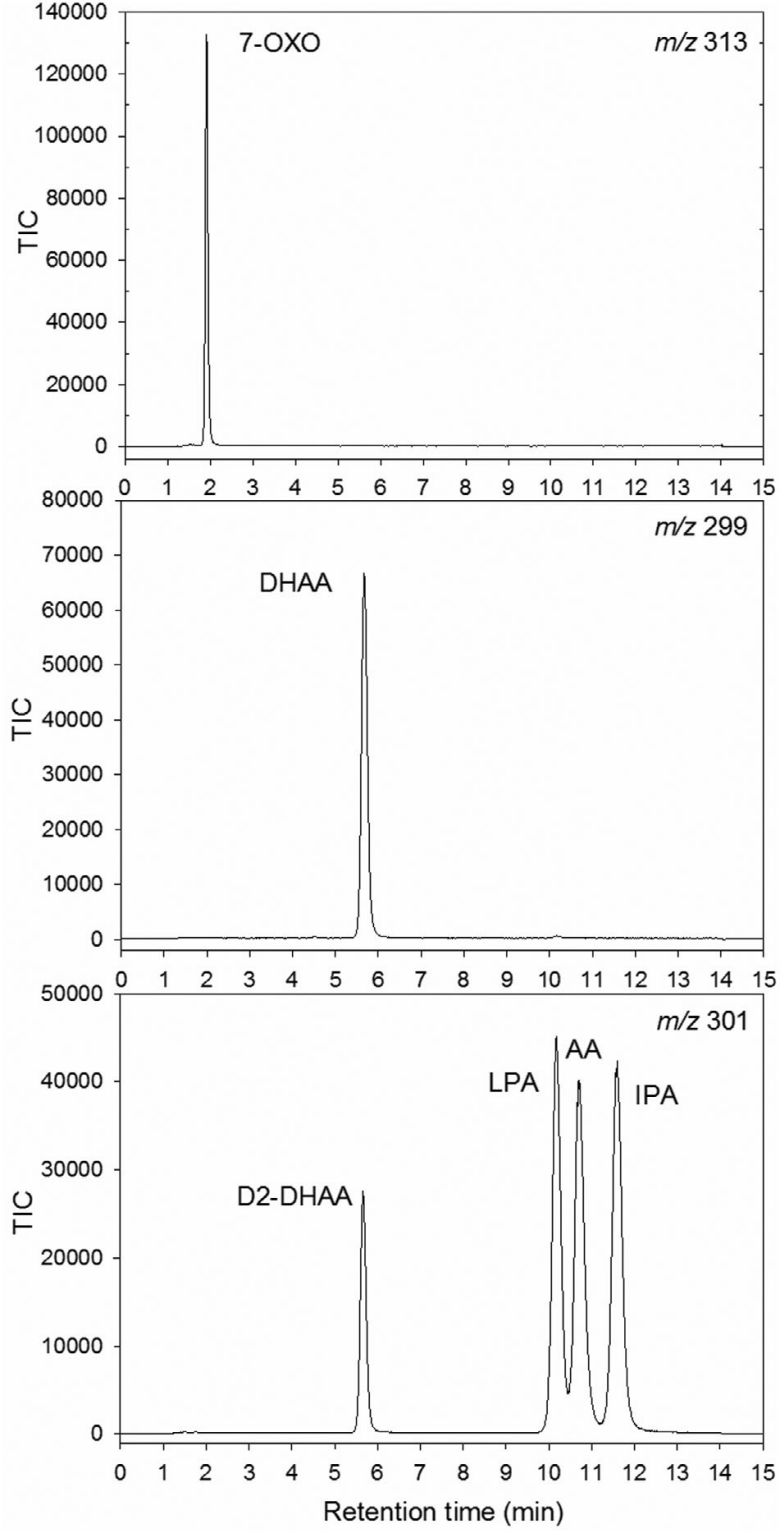
Total ion chromatograms (TICs) of the selected ion reactions (SIR) *m/z* 299, 301, and 313 of a standard solution containing 500 ng mL^−1^ of the resin acids: 7‐OXO, DHAA, LPA, AA, and IPA, and 250 ng mL^−1^ D2‐DHAA.

### 3.3 Method Validation

The method was validated for qualitative and quantitative determination of resin acids in softwood sawmill dust samples. First, the purity of each resin acid was investigated prior to the method validation. Contributions of DHAA and IPA from the AA stock solution (0.018 ng DHAA/ng AA and 0.016 ng IPA/ng AA) and IPA from the LPA stock solution (0.029 ng IPA/ng LPA) were added to the calibration standard concentrations of DHAA and IPA, respectively.

Second, the retention time of one of the major resins (PA) was determined by injecting a standard solution of PA in methanol. In the optimized method, PA had the same retention time as IPA; however, the MS signal intensities of PA and IPA were almost identical. IPA was therefore used for quantification of the sum of the resin acids IPA and PA in the rest of the study. In the method validation, 2 mL of the calibration standard solutions were added to 4 mL glass sample vials containing an unexposed 37 mm PVC (5 µm) filter and subjected to the same sample preparation as the real samples and sample blanks. The total method was validated in the range of 0.04–4 ng, which corresponds to the range of 10–1000 ng per sample. The method was linear within the investigated concentration range, with a coefficient of correlation better than 0.9995 for all analytes. The within‐ and between‐assay precisions were established by injecting six sets of samples at four levels (5, 25, 250, and 500 ng mL^−1^; injection volume of 8 µL) within 1 and 6 days, respectively, by the same analyst. The within‐assay (*n* = 6) and between‐assay precisions were in the range 0.2%–5.3% and 0.1%–4.8%, respectively, as summarized in Table 1. The within‐ and between‐assay RSDs of retention times were below 0.7%, and the column efficiency remained invariant throughout the study. The same analytical column was used throughout the study, and identical chromatographic performance was observed by replacing the analytical column with a new column. All calibration, validation, and real sample solutions were stable for at least one month when stored in the dark at ‐20°C. Assuming 100% extraction recovery, the mass limits of detection (LODs) were 5.5, 6.3, 5.3, 5.3, and 5.3 ng per sample for 7‐OXO, DHAA, LPA, AA, and IPA, respectively. Thus, the LODs obtained by using our method were comparable to the LODs obtained by Axelsson et al. [20], apart from the LOD obtained for 7‐OXO, which was tenfold higher using our method.

**TABLE 1:** Within‐ and between‐assay precisions of the total method (*n* = 6, injection volume 8 µL).

Within‐assay precision (%)					
Concentration level	Analytes				
(ng mL^−1^)	7‐OXO	DHAA	LPA	AA	IPA
5	1.5	1.8	2.8	5.3	3.5
25	1.9	1.0	1.2	1.8	3.0
50					
125					
250	2.1	0.5	2.7	2.6	2.7
375					
500	0.9	0.2	3.1	2.9	2.6

Between‐assay precision (%)					
Concentration level	Analytes				
(ng mL^−1^)	7‐OXO	DHAA	LPA	AA	IPA
5	1.7	2.0	3.9	3.1	4.8
25	2.5	0.6	2.5	1.2	3.2
50					
125					
250	1.2	0.4	4.3	3.7	4.6
375					
500	1.6	0.1	3.0	3.7	3.6

### 3.4 Determination of Resin Acids in Sawmill Dust Sample Extracts

To demonstrate the suitability of the optimized method, the resin acids DHAA, LPA, AA, the sum of the two resin acids IPA and PA, and the oxidation product of AA, 7‐OXO, were determined in six sawmill dust sample extracts. Sawmill dust sample extracts with concentrations of resin acids outside the calibration range of the optimized method were diluted with the d_2_‐DHAA solution. The amounts of resin acids determined in the six sawmill dust sample extracts were previously published in Straumfors et al. [12] as part of a larger dataset and are summarized in Table 2. To view these results from an occupational exposure perspective, total resin acid (TRA) concentrations were calculated and included in the table. TRA concentrations are used as a basis for occupational exposure assessment of rosin‐based solder flux fumes [25]. The British occupational exposure limit (OEL) for TRA is 0.05 mg m^−3^ (8‐h time‐weighted average) [29], while the threshold limit value (TLV) recommended by the American Conference of Governmental Industrial Hygienists (ACGIH) is 1 µg m^−3^ (8‐h time‐weighted average) [30]. Two of the sawmill dust samples contained TRA in higher concentrations than the British OEL, while all except one sawmill dust sample extract contained TRA concentrations higher than the ACGIH TLV.

**TABLE 2:** Concentration of individual resin acids and total resin acids (TRA) in six sawmill dust samples.

Sample	7‐OXO	DHAA	LPA	AA	IPA	TRA
	(ng m^−3^)					
1^ab^	32	442	241	467	277	1460
2^ab^	94	1148	276	620	590	2728
3^ab^	21	170	23	69	74	357
4^abc^	7117	21 956	6649	14 997	10 737	61 456
5^abc^	1648	28 059	17 984	29 868	17 847	95 407
6^abd^	1006	6997	2238	6393	2746	19 381

^a^ Air volumes samples (m^3^): 1: 0.81, 2: 0.81, 3: 0.69, 4: 0.87, 5: 0.80, and 6: 0.81.

^b^ Expanded uncertainties for the combined air sampling and analytical method were estimated to 22% for 7‐OXO and DHAA, and 23% for LPA, AA, and IPA.

^c^ Sawmill dust sample extract diluted 100 times prior to analysis.

^d^ Sawmill dust sample extract diluted 10 times prior to analysis.

Figure 3A,B shows the total ion chromatograms (TICs) of the selected ion reactions (SIR) m/z 299, 301, and 313 of two sawmill dust sample extracts. A broad AA peak was observed in both sawmill dust sample extracts. In addition, an extra peak maximum was also observed in the tailing of the AA peak in one of the sample extracts (see Figure 3B). This indicated that AA coeluted with at least one extra resin acid present in the sawmill dust sample extracts. According to the literature (Fengel and Wegener [5]), both Scots pine and Norway spruce contain the resin acids NA and PALA. The share of NA and PALA in Norway spruce has been estimated at 6.5%–11.0% and 13.5%–17.5% of the total amount of resin acids, respectively [5]. For Scots pine, the share of NA and PALA has been estimated at 11.1% and 15.1% of the total amount of resin acids, respectively. Thus, additional analytical standards of the resin acids NA and PALA were purchased and analysed, showing that both NA and PALA coeluted with AA. NA eluted at almost the same retention time as AA (+ 0.06 min), while the PALA peak maximum was approximately 0.2 min after the AA peak maximum, providing a strong indication that the resin acid observed in the tail of the AA peak in some of the sawmill dust sample extracts was PALA. To our knowledge, coelution of NA and PALA with AA was not included in the study by Axelsson et al. [20] analysing occupational total dust samples collected during wood pellet production. The identical molecular masses of the resin acids IPA, AA, LPA, NA, PA, and PALA make it impossible to determine the individual resin acids by MS without a chromatographic separation in advance. Optimal conditions for chromatographic separation resulted in suboptimal conditions for formation of fragmentation ions, and identification of individual resin acids from the fragmentation pattern was not possible using the proposed method. However, the signal intensities of PALA and NA were almost identical to AA (<10% difference). Likewise, the signal intensity of PA was almost identical to IPA. Thus, AA and IPA were used in this study for quantification of the sum of the resin acids AA, NA, and PALA, and IPA and PA, respectively.

**FIGURE 3:**
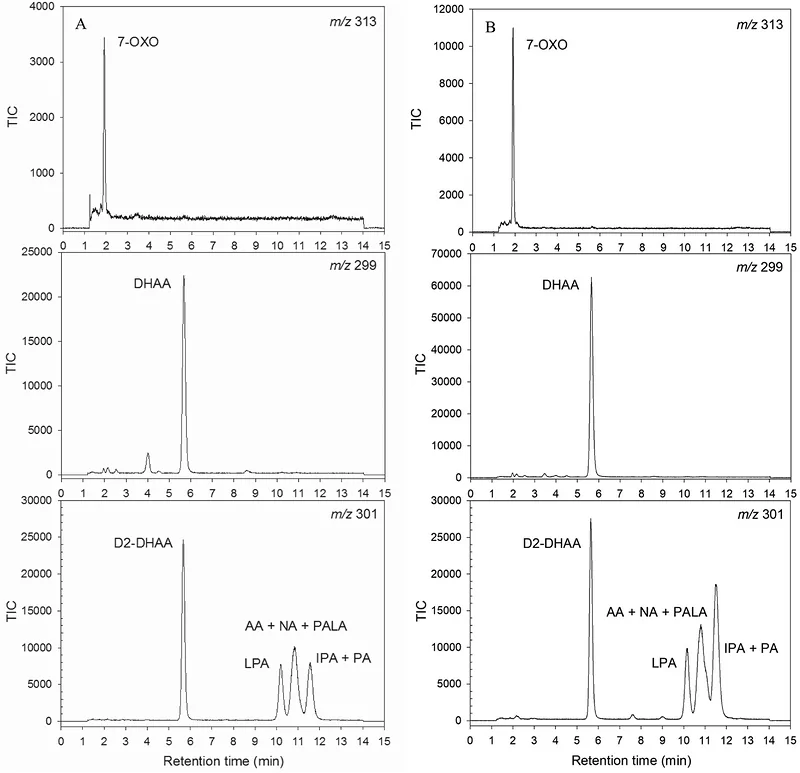
Total ion chromatograms (TICs) of the selected ion reactions (SIR) *m/z* 299, 301, and 313 of filter extracts containing (A) sample 1 and (B) sample 2, as listed in Table 2.

## 4 Conclusions

In this study, we have demonstrated a versatile, rapid, robust, sensitive, and selective LC‐APCI‐MS method with an improved separation of resin acids compared with earlier published LC methods. This LC‐APCI‐MS method provides less information than a GC‐MS or GC‐FID method about the exact resin acid composition due to poorer separation of resin acids. However, LC‐APCI‐MS is an ideal technique for rapid routine monitoring of occupational resin acid exposure, with minimum sample preparation. Thus, this versatile method can be used to determine airborne occupational exposure to resin acids in personal samples from Norwegian sawmill workers.

## Author Contributions

**Raymond Olsen**: writing – original draft preparation; conceptualization; funding acquisition; project administration; methodology; formal analysis; validation; visualization. **Ingrid Bakke**: writing – review & editing. The authors thank Rachel Louise Hasting for language editing.

## Conflicts of Interest

The authors declare no conflicts of interest.

## Funding

Financial contribution to the study was received from the Research Council of Norway, project no. 218232/H20.

## Data Availability

The data supporting this study's findings are available from the corresponding author, Ingrid Bakke, upon reasonable request.

## References

[1] D. A. Enarson and M. Chan‐Yeung , “Characterization of Health Effects of Wood Dust Exposures,” American Journal of Industrial Medicine 17 (1990): 33–38, 10.1002/ajim.4700170107. 2407114

[2] P. Demers , K. Teschke , and S. Kennedy , “What to Do About Softwood? A Review of Respiratory Effects and Recommendations Regarding Exposure Limits,” American Journal of Industrial Medicine 31 (1997): 385–398, 10.1002/(SICI)1097-0274(199704)31:4<385::AID-AJIM3>3.0.CO;2-V. 9093652

[3] T. Estlander , R. Jolanki , K. Alanko , and L. Kanerva , “Occupational Allergic Contact Dermatitis Caused by Wood Dusts,” Contact Dermatitis 44 (2001): 213–217, 10.1034/j.1600-0536.2001.044004213.x. 11260236

[4] International Agency for Research on Cancer (IARC) , “ A Review of Human Carcinogens Part C: Arenic, Metals, Fibres, and Dusts (IARC Monographs on the Evaluation of Carcinogenic Risk of Chemicals to Humans, Monograph, vol. 100C) ,” IARC (2012).

[5] D. Fengel and G. Wegener , Wood—chemistry, Ultrastructure, Reactions (Kessel Verlag, 2003), 190.

[6] N. Joye and R. Lawrence , “Resin Acid Composition of Pine Oleoresins,” Journal of Chemical & Engineering Data 12 (1967): 279–282, 10.1021/je60033a034.

[7] S. Prinz , U. Müllner , J. Heilmann , et al., “Oxidation Products of Abietic Acid and Its Methyl Ester,” Journal of Natural Products 65 (2002): 1530–1534, 10.1021/np010656l. 12444672

[8] E. Ehrin and A.‐T. Karlberg , “Detection of Rosin (colophony) Components in Technical Products Using an HPLC Technique*,” Contact Dermatitis 23 (1990): 359–366, 10.1111/j.1600-0536.1990.tb05172.x. 2096025

[9] A.‐T. Karlberg , “Air Oxidation Increases the Allergic Potential of Tall‐oil Rosin. Colophony Contact Allergens Also Identified in Tall‐oil Rosin,” American Journal of Contact Dermatitis 2 (1991): 43.

[10] P. A. Botham , D. Lees , H. P. A. Illing , and T. Malmfors , “On the Skin Sensitisation Potential of Rosin and Oxidised Rosin,” Regulatory Toxicology and Pharmacology 52 (2008): 257–263, 10.1016/j.yrtph.2008.05.013. 18586064

[11] U. Nilsson , N. Berglund , F. Lindahl , et al., “SPE and HPLC/UV of Resin Acids in Colophonium‐Containing Products,” Journal of Separation Science 31 (2008): 2784–2790, 10.1002/jssc.200800210. 18666177

[12] A. Straumfors , M. Corbin , D. McLean , et al., “Exposure Determinants of Wood Dust, Microbial Components, Resin Acids and Terpenes in the Saw‐ and Planer Mill Industry,” Annals of Work Exposures and Health 64 (2020): 282–296, 10.1093/annweh/wxz096. PMC7064270 31942929

[13] A. Straumfors , R. Olsen , H. L. Daae , et al., “Exposure to Wood Dust, Microbial Components, and Terpenes in the Norwegian Sawmill Industry,” Annals of Work Exposures and Health 6 (2018): 674–688, 10.1093/annweh/wxy041. PMC6037117 29878039

[14] A. M. Harju , P. Kainulainen , M. Venäläinen , M. Tiitta , and H. Viitanen , “Differences in Resin Acid Concentration Between Brown‐rot Resistant and Susceptible Scots Pine Heartwood,” Holzforschung 56 (2002): 479–486, 10.1515/HF.2002.074.

[15] K. Li , A. Serreqi , C. Breuil , and J. Saddler , “Quantification of Resin Acids in CTMP (chemithermomechanical pulping) Effluents Using an Enzyme‐linked Immunosorbent Assay,” Water Science and Technology 35 (1997): 93–99, 10.2166/wst.1997.0491.

[16] B. Lee , H. Ong , D. Koh , and C. Ong , “High‐performance Liquid Chromatographie Method for Determination of Dehydroabietic and Abietic Acids, the Skin Sensitizers in Bindi Adhesive,” Journal of Chromatography A 685 (1994): 263–269, 10.1016/0021-9673(94)00691-1. 9129324

[17] B. Lee , D. Koh , H. Ong , and C. Ong , “High‐performance Liquid Chromatographic Determination of Dehydroabietic and Abietic Acids in Traditional Chinese Medications,” Journal of Chromatography A 763 (1997): 221–226, 10.1016/S0021-9673(96)00901-6. 9129324

[18] A. Latorre , A. Rigol , S. Lacorte , and D. Barceló , “Comparison of Gas Chromatography–Mass Spectrometry and Liquid Chromatography–Mass Spectrometry for the Determination of Fatty and Resin Acids in Paper Mill Process Waters,” Journal of Chromatography A 991 (2003): 205–215, 10.1016/S0021-9673(03)00217-6. 12741600

[19] K. Hagström , S. Axelsson , H. Arvidsson , I.‐L. Bryngelsson , C. Lundholm , and K. Eriksson , “Exposure to Wood Dust, Resin Acids, and Volatile Organic Compounds During Production of Wood Pellets,” Journal of Occupational and Environmental Hygiene 5 (2008): 296–304, 10.1080/15459620801957225. 18322870

[20] S. Axelsson , K. Eriksson , and U. Nilsson , “Determination of Resin Acids During Production of Wood Pellets—A Comparison of HPLC/ESI‐MS With the GC/FID MDHS 83/2 Method,” Journal of Environmental Monitoring 13 (2011): 2940, 10.1039/c0em00543f. 21874165

[21] D. W. McMartin , K. M. Peru , J. V. Headley , M. Winkler , and J. A. Gillies , “Evaluation of Liquid Chromatography–negative Ion Electrospray Mass Spectrometry for the Determination of Selected Resin Acids in River Water,” Journal of Chromatography A 952 (2002): 289–293, 10.1016/S0021-9673(02)00106-1. 12064541

[22] P. Demers , K. Teschke , H. Davies , S. Kennedy , and V. Leung , “Exposure to Dust, Resin Acids, and Monoterpenes in Softwood Lumber Mills,” American Industrial Hygiene Association Journal 61 (2000): 521–528, 10.1080/15298660008984564. 10976682

[23] P. A. Smith , D. R. Gardner , D. B. Drown , G. Downs , W. W. Jederberg , and K. Still , “Detection of Resin Acid Compounds in Airborne Particulate Generated From Rosin Used as a Soldering Flux,” American Industrial Hygiene Association Journal (1997): 868–875, 10.1080/15428119791012207. 9425647

[24] P. A. Smith , D. R. Gardner , D. B. Drown , W. W. Jederberg , and K. Still , “Oxidized Resin Acids in Aerosol Derived From Rosin Core Solder,” American Industrial Hygiene Association Journal (1998): 889–894, 10.1080/15428119891011063. 9866169

[25] Health and Safety Executives , “ MDHS 83/3 Resin Acids in Rosin (colophony) Solder Flux Fume: Laboratory Method Using Gas Chromatography ,” HSE (2006).

[26] F. Dethlefs , K.‐O. Gerhardt , and H.‐J. Stan , “Gas Chromatography/Mass Spectrometry of 13 Resin Acids as Their PFB Esters,” Journal of Mass Spectrometry 31 (1996): 1163–1168, 10.1002/(SICI)1096-9888(199610)31:10<1163::AID-JMS406>3.0.CO;2-3.

[27] T. McGinnis , “Quantitative Determination of Fatty and Resin Acids in Kraft Black Liquors as Their Trimethylsilyl Derivatives by Gas Chromatography,” Journal of Chromatography A 829 (1998): 235–249, 10.1016/S0021-9673(98)00829-2.

[28] C. Nolte , J. Schauer , G. Cass , and B. Simoneit , “Trimethylsilyl Derivatives of Organic Compounds in Source Samples and in Atmospheric Fine Particulate Matter,” Environmental Science & Technology 36 (2002): 4273–4281, 10.1021/es020518y. 12387398

[29] Safety Executive . EH40/20005 Workplace Exposure Limits (HSE Books, 2005).

[30] American Conference of Governmental Industrial Hygienists , “ 2025 TLVs and BEIs: Threshold Limit Values for Chemical Substances and Physical Agents and Biological Exposure Indices ,” ACGIH, (2025).

